# The effect of mobile phone text message reminders on health workers’ adherence to case management guidelines for malaria and other diseases in Malawi: lessons from qualitative data from a cluster-randomized trial

**DOI:** 10.1186/s12936-018-2629-2

**Published:** 2018-12-19

**Authors:** Blessings N. Kaunda-Khangamwa, Laura C. Steinhardt, Alexander K. Rowe, Austin Gumbo, Dubulao Moyo, Humphreys Nsona, Peter Troell, Dejan Zurovac, Don Mathanga

**Affiliations:** 1 Malaria Alert Centre (MAC), Communicable Disease Action Centre, University of Malawi College of Medicine, Blantyre, Malawi; 2 School of Public Health, The University of Witwatersrand, Johannesburg, South Africa; 3 Malaria Branch, Division of Parasitic Diseases and Malaria, Center for Global Health, Centers for Disease Control and Prevention, Atlanta, GA USA; 4 National Malaria Control Programme, Ministry of Health, Lilongwe, Malawi; 5 Integrated Management of Childhood Diseases Program, Ministry of Health, Lilongwe, Malawi; 6 President’s Malaria Initiative, Malaria Branch, Division of Parasitic Diseases and Malaria, Centers for Disease Control and Prevention, Lilongwe, Malawi; 7 KEMRI-Wellcome Trust-Research Programme, Nairobi, Kenya; 8 Centre for Tropical Medicine and Global Health, University of Oxford, Oxford, UK

**Keywords:** mHealth, Childhood illness, Malaria, Diarrhoea, Pneumonia, Case management, Malawi, Qualitative, Text message

## Abstract

**Background:**

Mobile health (mHealth), which uses technology such as mobile phones to improve patient health and health care delivery, is increasingly being tested as an intervention to promote health worker (HW) performance. This study assessed the effect of short messaging services (SMS) reminders in a study setting. Following a trial of text-message reminders to HWs to improve case management of malaria and other childhood diseases in southern Malawi that showed little effect, qualitative data was collected to explore the reasons why the intervention was ineffective and describe lessons learned.

**Methods:**

Qualitative data collection was undertaken to lend insight into quantitative results from a trial in which 105 health facilities were randomized to three arms: (1) twice-daily text-message reminders to HWs, including clinicians and drug dispensers, on case management of malaria; (2) twice-daily text-message reminders to HWs on case management of malaria, pneumonia and diarrhoea; and, (3) a control arm. In-depth interviews were conducted with 50 HWs in the intervention arms across seven districts. HWs were asked about acceptability and feasibility of the text-messaging intervention and its perceived impact on recommended case management. The interviews were recorded, transcribed and translated into English for a thematic and framework analysis. Nvivo 11 software was used for data management and analysis.

**Results:**

A total of 50 HWs were interviewed at 22 facilities. HWs expressed high acceptance of text-message reminders and appreciated messages as job aids and practical reference material for their day-to-day work. However, HWs said that health systems barriers, including very high outpatient workload, commodity stock-outs, and lack of supportive supervision and financial incentives demotivated them, limited their ability to act on messages and therefore adherence to case management guidelines. Drug dispensers were more likely than clinicians to report usage of text-message reminders. Despite these challenges, nearly all HWs expressed a desire for a longer duration of the SMS intervention.

**Conclusions:**

Text-message reminders to HWs can provide a platform to improve understanding of treatment guidelines and case management decision-making skills, but might not improve actual adherence to guidelines. More interaction, for example through targeted supervision or two-way technology communication, might be an essential intervention component to help address structural barriers and facilitate improved clinical practice.

## Background

Malaria remains a significant cause of morbidity and mortality in Malawi. National surveys indicate persistently high parasitaemia prevalence in children under 5 years old, estimated to be 28% in 2012, 33% in 2014, and 24% in 2017 [1–3]. There are an estimated 6.2 million malaria cases per year, accounting for 30% of outpatient visits to health facilities across all ages, although diagnosis and treatment practices show gaps in health worker (HW) performance [4]. Health facility surveys in Malawi, undertaken before the widespread introduction of malaria rapid diagnostic tests (RDTs), indicated that only two-thirds of patients with suspected malaria were correctly treated [5]. RDTs have the potential to improve malaria diagnosis and treatment, but only if they are used consistently. Health facility surveys conducted in southern Malawi after the scale-up of RDTs found that three-quarters of patients were correctly tested for malaria, but only 6% of patients with suspected severe malaria were correctly treated and referred [6]. After malaria, the leading causes of child mortality in Malawi include pneumonia and diarrhoea, accounting for 13 and 7%, respectively, of deaths among children 1–59 months [7, 8]. These diseases, however, are often under-diagnosed and under-treated or mismanaged, ultimately increasing mortality [9, 10].

Previous studies have shown that mobile health (mHealth) has the potential to improve healthcare, including HW adherence to case management guidelines [11–14]. The World Health Organization (WHO) defines mHealth as medical and public health practice supported by mobile devices such as phones, patient monitoring devices or tablets [15]. One mHealth application that has the potential to improve HW performance involves sending short message alerts, clinical guidelines and learning materials to HWs (medical assistants, clinicians, health surveillance assistants, pharmacy technicians, nurse aides, drug dispensers) [14, 16, 17]. A recent trial in Kenya indicated that text-message reminders could substantially improve HW case management of malaria in children [14]. To expand the evidence around text messaging or short messaging services (SMS) reminders for promoting HW behaviour change in low-income settings, a trial was undertaken in 2015–2016 in southern Malawi to evaluate the effectiveness of sending SMS reminders to HWs’ mobile phones to improve the management of malaria, pneumonia and diarrhoea at health facilities. The trial in Malawi did not find much effect from text messages (overall a non-significant 4%-point improvement in correct malaria case management), with improvements in malaria, pneumonia and diarrhoea case management no better in the intervention arms than in the control (Steinhardt et al., pers. comm.). This follow-up study used qualitative methods to describe HW perceptions of the messages received, possible mechanisms of action and potential challenges to acting on the SMS reminders with the overarching goal of understanding the reasons why the intervention was ineffective and elucidate lessons learned.

## Methods

### Study setting

This study took place in southern Malawi, where parasitaemia prevalence among children 6–59 months of age was 26% in 2017, similar to the national prevalence of 24% [3]. Thirty-seven per cent of children with fever in Malawi are brought to a public sector provider, and an additional 4% are taken to a Christian Health Association of Malawi (CHAM) facility, with 14% taken to other sources and 46% not taken for care [3]. Malawi guidelines stipulate that febrile patients be tested for malaria and treated accordingly. Malaria case management refresher training occurs on a regular basis, with the most recent training occurring in southern districts in late 2014. HWs are trained in integrated management of childhood illness (IMCI) for children under 5 years of age during pre-service training; the latest national-level IMCI refresher training last took place in 2010.

The IMCI package provides the standard of care at health facilities for common childhood illnesses, including malaria, diarrhoea and pneumonia. HWs diagnose and treat both uncomplicated and complicated cases of the illnesses mentioned earlier according to national guidelines based on IMCI algorithms, and refer severe malaria and severe pneumonia cases to inpatient settings of higher-level facilities. HWs are supervised periodically ranging from monthly, quarterly or annually depending on resource constraints to ensure good implementation of routine practices.

### Main trial design

This study is part of a larger cluster-randomized, controlled trial with pre- and post-intervention evaluations, with health facilities as the clusters (units of randomization). The objectives of the trial were to evaluate the effectiveness of SMS reminders to improve case management and quality of care for malaria, diarrhoea and pneumonia. Altogether, 105 health facilities operated by the Government of Malawi or CHAM within the southern region of Malawi were randomized to one of three study arms: (1) SMS reminders to HWs on the correct management of malaria (patients of all ages); (2) SMS reminders to HWs on the correct management of malaria (patients of all ages) and of diarrhoea and pneumonia (children under 5); and, (3) a control arm where HWs received no messages. All HWs in the intervention arm providing outpatient care, as well as HWs dispensing drugs and, therefore, tasked with patient counselling about medication administration and adherence, were targeted to receive SMS reminders twice a day for 6 months. In Malawi, drug dispensers are lower-level cadre HWs, in many cases with minimal training; in some cases, patient attendants, ground labourers and security guards with on-the-job training play the role of drug dispensers [18].

### SMS intervention

The intervention involved a one-way communication of SMS reminders about recommended case management to clinicians (medical assistants, clinical officers, nurses, and others) providing outpatient care and to drug dispensers working in the pharmacy and providing counselling on medications. HWs were asked for their written consent to receive messages and participate in a baseline health facility survey, and their mobile phone numbers were collected. Text-message reminders were sent to HWs twice a day in the intervention groups for 6 months between April and October 2015. In arm 1, the malaria-only arm, 10 unique messages (in English) on malaria case management for patients of all ages were sent to HWs each week (see Arm 1 messages in Table 1). Messages reminded HWs to assess all patients for fever/history of fever, test all patients with fever for malaria, not to give anti-malarials for a negative malaria test, and also instructed HWs about first- and second-line treatments and dosing for uncomplicated and severe malaria, and on key dispensing and counselling messages (i.e., to give the first dose at the facility and complete all doses). The same messages were repeated for 26 weeks; each message was accompanied by a unique proverb, in either English or Chichewa (Malawi’s local language), which was designed to keep HWs interested in reading the reminders. There was a 160-character limit on the messages.

**Table 1:** Malaria messages sent to intervention Arm 1

Timing	Arm 1: Malaria only
Mon a.m.	“Check ALL patients with fever or history of fever for signs of severe malaria! If any severe sign TREAT & REFER urgently!” Better be safe than sorry
Mon p.m.	“Ask ALL patients about fever, take temperature and check other malaria signs and symptoms.” Where there is smoke there is fire
Tues a.m.	“Test for malaria ALL children < 5 and preg women with fever. Also test ALL patients ≥ 5 with fever + 1 symptom.” Look before you leap
Tues p.m.	“For febrile patients without severe signs TREAT for malaria ONLY if test is positive—do NOT treat negatives for malaria!” A word to the wise is sufficient
Wed a.m.	“When malaria test is NEGATIVE check for other causes; if none found give antipyretic and ask patient to return if fever persists!” Persistent work triumphs
Wed p.m.	“For uncomplicated malaria 1st line Rx is LA; 2nd line is ASAQ. For children < 5 kg and in 1st preg trimester give quinine + clindamycin.” Do the right thing
Thurs a.m.	“New pre-referral Rx for severe malaria is IM artesunate; if not available use IM quinine; if quinine O/S use rectal artesunate.” Never too old to learn
Thurs p.m.	“Prescribe LA based on WEIGHT: 1 × 6 for 5–14 kg; 2 × 6 for 15–24 kg; 3 × 6 for 25–34 kg; 4 × 6 for ≥ 35 kg.” A goal without a plan is just a wish
Fri a.m.	“Give FIRST LA DOSE to ALL patients with uncomplicated malaria at FACILITY even if on an empty stomach!” To be willing is to be able
Fri p.m.	“Advise ALL patients to take 2nd LA dose after 8 h, then every 12 h to complete all 6 doses even if they feel better!” Say little but say it well

In Arm 2, HWs received the malaria messages 1 week, and these alternated with pneumonia and diarrhoea messages for children under 5 years (including a proverb) for the 26 weeks. Pneumonia and diarrhoea messages reminded HWs to check for danger signs, how to diagnose uncomplicated pneumonia (i.e., counting respiratory rate in children with a cough or difficult breathing) and severe pneumonia, and instructed them on correct treatments for pneumonia and diarrhoea (see Arm 2 messages in Table 2). Messages were based on the IMCI guidelines in Malawi, on which HWs are trained [19]. Baseline and endline health facility surveys were conducted to assess the effect of the messages on HW practices during patient consultations for key aspects of case management of malaria, pneumonia and diarrhoea. Methods and results of the main trial are presented elsewhere (see Steinhardt et al., pers. comm.).

**Table 2:** Malaria only and pneumonia, diarrhoea messages and proverbs posted on alternate weeks to Arm 2 health workers

sn Timing	Arm I: Malaria only	Arm 2: Malaria, pneumonia, and diarrhea
Mon a.m.	“Check ALL patients with fever or history of fever for signs of severe malaria! If any severe sign TREAT & REFER urgently!” Better be safe than sorry	“Check ALL sick children for any DANGER sign-if unable to drink OR lethargic OR vomit everything OR convulsions TREAT & REFER!” Well begun is half done
Mon p.m.	“Ask ALL patients about fever, take temperature and check other malaria signs and symptoms.” Where there is smoke there is fire	“Check ALL sick children for danger signs! Check ALL for fever, cough, difficult breathing, diarrhea, pallor, ear and other problems!” The seeker is the finder
Tues a.m.	“Test for malaria ALL children < 5 and preg women with fever. Also test ALL patients ≥ 5 with fever + 1 symptom.” Look before you leap	“For ALL children with cough or difficult breathing COUNT BREATHS in 1 min & look for chest in-drawing and stridor!” Actions speak louder than
Tues p.m.	“For febrile patients without severe signs TREAT for malaria ONLY if test is positive—do NOT treat negatives for malaria!” A word to the wise is sufficient	“Child has PNEUMONIA IF breath count is FAST: over 60 if less than 2 months, over 50 if 2–12 mos. or over 40 if 12–59 mos.” Never too old to learn
Wed a.m.	“When malaria test is NEGATIVE check for other causes; if none found give antipyretic and ask patient to return if fever persists!” Persistent work triumphs	“Child with cough or difficult breathing has SEVERE PNEUMONIA if any danger sign, chest in-drawing or stridor present-TREAT & REFER!” Knowledge is power
Wed p.m.	“For uncomplicated malaria 1st line Rx is LA; 2nd line is ASAQ. For children < 5 kg and in 1st preg trimester give quinine + clindamycin” Do the right thing	“Child does NOT have pneumonia if breath count is NOT fast and NO danger/severe sign—treat for cold; do NOT give antibiotic!” Things don’t change; we change
Thurs a.m.	“New pre-referral Rx for severe malaria is IM artesunate; if not available use IM quinine; if quinine 0/S use rectal artesunate.” Never too old to learn	“For pneumonia 1st line Rx is amoxicillin and 2nd line is erythromycin. For SEVERE pneumonia treat with IM X-pen & REFER!” It works if you work it
Thurs p.m.	“Prescribe LA based on WEIGHT: 1 × 6 for 5–14 kg; 2 × 6 for 15–24 kg; 3 × 6 for 25–34 kg; 4 × 6 for ≥ 35 kg.” A goal without a plan is just a wish	“Give ALL children FIRST antibiotic dose at facility, explain dosing at home and advise to finish all doses even if feel better!” A little late is too late
Fri a.m.	“Give FIRST LA DOSE to ALL patients with uncomplicated malaria at FACILITY even if on an empty stomach!” To be willing is to be able	“Give ALL children with diarrhea ORS, zinc and advice on extra fluids; give antibiotics ONLY if blood in stool!” First things first
Fri p.m.	“Advise ALL patients to take 2nd LA dose after 8 h, then every 12 h to complete all 6 doses even if they feel better!” Say little but say it well	“Assess dehydration in child with diarrhea; if some dehydration give ORS + 1st dose zinc at FACILITY; if severe give IV fluid or REFER!” Willingness is the key

### Qualitative data collection

An explanatory sequential method for qualitative data collection [20] was used, meaning that the quantitative data results informed qualitative research sampling and data collection. From the 70 facilities in the intervention arms, 28 health facilities (14 per arm) were purposively selected to reflect a range of settings (e.g., health centre vs hospital), hard to reach/easily accessible sites and peri-urban/rural residence, and ‘intervention response’ measured by changes in case-management practices from baseline, as end-line health facility survey results were available prior to qualitative data collection. Of the 28 health facilities, four district hospitals were included (2 per intervention arm). The remaining facilities were health centres: two in urban areas in Arm 2, 2 in peri-urban areas in Arm 1, and 20 in rural areas (10 in Arm 1, and 10 in Arm 2).

All HWs providing outpatient care or dispensing drugs (n = 81) at the 28 sampled facilities during the data collection team’s baseline visit were eligible for in-depth interviews (IDIs). Objectives of the IDIs were to gain an in-depth understanding of how SMS reminders were perceived and to elucidate what effects the SMS reminders had, in particular, the reasons they were not very effective in changing HW case management practices. Prior to the interviews, a phone call and a site visit were made to confirm HWs’ availability and participation in the study.

IDIs were conducted by a senior social scientist (BKK) in collaboration with five experienced qualitative research assistants. The interviews were held at the participants’ health facilities in the afternoon hours when patient loads are generally lighter to ensure HW full participation. Written informed consent was obtained from HWs participating in the study.

Semi-structured interview guides with open-ended questions were used, and interviewers ensured that all the questions and topics on the interview guide were covered thoroughly. The research team also recorded notes during the discussion to capture non-verbal types of communication. Topics covered included perceptions of the SMS intervention, modalities of SMS intervention (timing, frequency, dialect, length, duration), acceptability and feasibility, impact of SMS reminders on case management, and challenges associated with diagnostic procedures (malaria, pneumonia, diarrhoea), supervision, monitoring, and adherence to IMCI guidelines. All interviews were digitally recorded and later transcribed for analysis.

The research team used both English and Chichewa, depending on HW preference during the face-to-face interviews. Dominant themes, understanding, and depth of responses were dependent on time and saturation (when no new information emerged from the data). Data collection took place from 23 January to 5 February, 2016. The study was approved by the CDC Institutional Review Board (IRB) as well as the College of Medicine Research Ethical Committee (COMREC) in Malawi (P11/13/1488).

### Data management and analysis

The IDIs were transcribed and translated into English for the few drug dispensers who elaborated their ideas in Chichewa. Interviewer notes from non-verbal behaviours were used to supplement the interview transcripts. The senior researcher and fieldwork team developed the initial coding framework guided by the study objectives, research questions and recurring themes.

A Nvivo 11 (QSR International, Victoria, Australia) computer software was used to code and analyse data using framework analysis [21]. Framework analysis allows data to be classified and organized according to key themes, concepts and emergent categories. The first author and the study investigators regularly shared coded notes and discussed the main themes and sub-themes to improve their understanding of key findings. Any differences of opinion on coding or data interpretation were discussed until consensus could be reached. The main themes and sub-themes were further refined as the authors familiarized themselves with more transcripts. Through an iterative process, research team members were able to analyse, chart and summarize data in preparation for interpretation. In the last phase of the analysis, coded data associated with main themes, e.g., action taken or not taken after receiving SMS reminders, adherence to case management and treatment guidelines, were extracted and provided as quotes.

## Results

Of the 81 HWs (40 in Arm 1 and 41 in Arm 2) targeted for interviews, 50 were interviewed, including 28 drug dispensers and 22 HWs (clinicians/medical assistants and nurses) across 22 facilities; 11 HWs had transferred to other facilities, 6 had gone back to school for additional training, two refused to be part of the qualitative study, two lived in hard-to-reach areas that study teams could not access due to the rains, and 10 never received the SMS reminders, despite consenting to receive them, due to various reasons such as a lost/stolen phone or having changed a phone number. These HWs worked at 22 of the 28 facilities targeted for interviews. Most interviews took place in English and lasted 30–90 min. A list of HWs who participated in the study is listed in Table 3.

**Table 3:** Health worker cadres interviewed, by intervention arm

Type of health worker	Arm 1: Malaria-only messages	Arm 2: Malaria, pneumonia, and diarrhoea messages
Clinicians		
Clinical officer	1	4
Medical assistant	8	6
Nurse midwife	1	2
Drug dispensers		
Health surveillance assistants (HSA)	4	5
Pharmacy technician	0	2
Pharmacy attendant	2	6
Hospital attendants	8	1

### Perceptions of the SMS intervention

The majority (45/50, 90%) of HWs in the study viewed the text-message intervention favourably. Some HWs were enthusiastic and viewed them as a “*likasa* (job aide)” “e-handbook,” “guiding tool,” “reminder,” or “alarm” that alerted and encouraged them to adhere to standards and guidelines as well as to improve their case-management skills and ultimately their interactions with patients. Some clinicians appreciated SMS reminders as providing reference materials when dealing with patients suffering from malaria, pneumonia and diarrhoea. Likewise, some clinicians/medical assistants/nurses printed the SMS reminders and placed them on the notice boards in their offices for easy reference when in doubt about classification or treatment during consultations. Others placed the phones on their working desks for easy accessibility as reference materials without drawing the attention of the patient whenever they forgot the common childhood management practices.

*I believe the messages helped me to change my attitude and approach on how to manage some cases. I changed a lot. For example, about malaria cases, we never used to test all suspected malaria cases…Each time, I saw the message in the morning, I would know that I have to start work. I feel the messages being on my phone, acted as a reference that was right in front of me, it helped my work and guaranteed the provision of quality case management.* (Arm 2, Medical Assistant)

Most clinicians were able to recall quotes on malaria and pneumonia guidelines, and a few recited some quotes on diarrhoea. However, 7/50, or 14% of HWs, mainly clinicians, were indifferent and argued that they already had prior knowledge through refresher training and exposure in medical school and did not appreciate the repetitions or the provisions of proverbs, which discouraged them from taking action after they received the SMS text messages.

Most drug dispensers copied the SMS reminders in a notebook and cardboard as job aids for easy reference as most of their phones could not hold many messages at a time. Others placed notes on a cardboard paper close to the dispensing window for accessibility and easy reference as they interacted with their patients. Text messages for drug dispensers acted as motivation as they provided knowledge on case management to provide better care services including better counselling to patients. All drug dispensers accepted the SMS intervention despite not being trained by case-management programmes. Most of them reported that the study had boosted their morale and confidence in handling malaria, pneumonia and diarrhoea cases.

*In the past patients with malaria, signs were being treated with anti*-*malaria drugs without being tested at the health facility. However, there was a significant improvement in handling patients when the text message reminders intervention was initiated. The text message reminders also helped the HWs to prescribe a right dosage of AL according to the patient’s weight and age. The text messages were evident on how a dosage of AL should be administered to patients. In the past children under five years were not given a full dosage of AL, however, now things have changed because we have been sensitized on how to administer a full dosage of antimalarials.* (Arm 2, Drug Dispenser)

### Modalities of SMS intervention (timing, frequency, dialect, length, proverbs)

#### Timing and frequency

Most HWs were content to receive the text messages twice a day, in the morning around 08.00 and after lunch around 13:30, 5 days a week. However, 15/50 or 30% of the respondents said they would have preferred receiving SMS during the weekends and a single message a day during the morning hours. Both the clinicians and drug dispensers claimed that receiving SMS during morning hours was most convenient as they were able to recall contents throughout the day. However, participants receiving malaria-only messages did not appreciate the repetitions over time. Others stopped reading the SMS in the subsequent weeks as they thought messages were being repeated and there was nothing new to learn. Some would open the message and delete it the moment they realized it was the same message they had received in the preceding weeks. Respondents in malaria/pneumonia/diarrhoea arm appreciated fewer SMS repetitions in the subsequent weeks.

#### Dialect

The majority of respondents were grateful that the messages were in English. The clinicians could not fathom the idea of the SMS being sent in the local language as that would distort the contents of case management/treatment messages. Others commented that SMS in Chichewa would become too long and difficult to understand. However, a few drug dispensers (5/28, or 18%) requested to have the SMS sent in Chichewa for better understanding and easy reference.

#### Length

All respondents appreciated the short length of the SMS that enabled them to read on their phone but still allowed for adequate information on a specific disease. Participants receiving malaria-only messages did not like the SMS repetitions. However, they were able to keep the messages in their phone folders for longer compared to those in Arm 2 who were forced to delete some as they were receiving more messages for malaria, pneumonia and diarrhoea.

#### Proverbs

There was mixed feedback on adding proverbs to pneumonia, malaria and diarrhoea SMS. Most HWs failed to recall any proverb quotes. A few reported that the proverbs were complementing the main case management message for the day (though this was not the intention behind them). The few clinicians who commented on the additional proverbs said they strengthened the SMS messages. However, others were indifferent and did not see the added value of having proverbs accompanying the case management guidelines.

### Duration of the intervention

Most of the respondents in the study did not know how long the SMS intervention ran, and a few clinicians reported that the intervention ran for 6 weeks (in reality, it lasted 6 months). For the 10 respondents who never received SMS reminder, they thought the SMS intervention study had not been implemented. Almost all respondents claimed the duration of the intervention was too short. Others wanted the SMS intervention to run from 6 months to a year with close supervision and monitoring. Some were sad that the SMS reminders stopped abruptly and that no one followed up to announce the closure of the study.

### Structural barriers to acting on messages

Despite the high SMS intervention acceptance, most HWs reported challenges related to feasibility or practicability of prompt action towards testing all malaria suspected cases, counting the respiratory rates of children with a cough or difficult breathing, as well as giving the first dose of anti-malarial or antibiotics in the health facility. Most HWs (45/50, or 90%) blamed the high workload, drug stock-outs, lack of supervision, monitoring, financial incentives, and feedback sessions that discouraged them from acting on the SMS reminders. Lack of financial incentives was mentioned in almost all interviews. Some HWs expected the study to hold monthly meetings where incentives such as an allowance including feedback sessions would be part of the intervention package. Similarly, half the HWs (25/50, or 50%) felt there was a need for more monitoring or supervision either on a monthly or quarterly basis to specifically reinforce the text messages. (Quantitative data indicated that at the 22 facilities with qualitative data collection, 15/30 (50%) of HWs interviewed at endline reported having a supervision visit in the past 6 months, with a mean and median of two visits among those receiving any supervision.)

*Maybe we should say it is because of workload here [hospital] since there is a lot of work going on around here. We receive referrals and most of the times we don’t have enough space to ask people to wait [observe them while taking drugs] while you are also busy administering drugs to different people. It is easy to do that at the health center because you see few people maybe not less than six people and it is not hard to do.* (Arm 2, Clinician)

*Well, the incentives can maybe come after the supervision. If the health workers are doing a good job, why not encourage them … but the most important issue to encourage is supervision*. (Arm 1, Clinician)

Most (20/50, or 66%) HWs were quick to refer to stock-outs of RDTs and drugs, unavailability of weighing scales, and the inability of health facilities to test all patients with fever on a daily basis. In case of stock-outs of anti-malarials, priority was given to children under the age of five, as adult dose packs of artemether–lumefantrine (AL) can be split. According to quantitative data collected several months before, 19/22 (86%) facilities where qualitative data collection subsequently took place had at least one type of dose pack of AL in stock on the day of the survey. Median numbers of stock-out days in the last 3 months were 0 (out of 90) for AL 1 × 6 and 2 × 6 and 15 and 19 for AL 3 × 6 and 4 × 6, respectively. Most (21/22, or 95%) facilities had at least one first-line antibiotic in stock the day of the survey, and 18/22 (86%) had oral rehydration solution (ORS) in stock the day of the endline survey (Steinhardt et al., pers. comm.). Some clinicians were overwhelmed by the workload and faced complaints from laboratory technicians and traditional authorities for causing caregivers to wait for a long time at health facilities to confirm a malaria test result. Among the selected OPDs at endline at the 22 health facilities where qualitative data collection took place, the endline quantitative data indicated mean and median daily caseloads of 97.0 and 80.5 patients, respectively (range, 17 to 250) (Steinhardt et al., pers. comm.).

***I:*** *Do you measure respiratory rate for the under*-*five?*

***R:*** *We have congestion here at the facility, and it is hard to measure the respiratory rate on every one… time is the problem. For example, when we have many people like the way it is now, we stay up to late afternoon hours and without taking lunch because of workload. So to examine everyone according to guidelines, it becomes difficult, and most of the times we measure the respiratory rate when a patient is critically ill than others. Yes, but I am prompted to select patients to conduct respiratory rate because of congestion. The congestion is always daily nowadays since November up to February, and we can see almost 500 patients per day.* (Arm 1, Clinician)

### Dispensing the first dose at the facility and correct dosing

Giving the first dose of anti-malarials or antibiotics at the facility was feasible with minimal monitoring, but it was a challenge at tertiary hospitals due to higher workloads. Both clinicians and drug dispensers in some facilities failed to support the standard of giving the first malaria dose at the health facility, as they did not have enough cups and water basins. Others referred the patients to drug dispensers to ensure that patients were indeed taking their first malaria dose at the facility. A few drug dispensers created a ‘malaria corner’ with a bucket, a few cups and took turns to do ‘directly observed therapy’ sessions. The malaria corner was popular during the first weeks of the SMS intervention but failed to continue in the subsequent weeks due to logistical challenges such as unavailability of cups and clean drinking water. Others blamed caregivers for refusing to give the first malaria dose at health facility fearing the child had not taken some food, while others reported lacking cups and HWs to monitor the whole process.

*Ok, the first dose of AL at the facility. In the past, we were told AL needs one to eat as a result when we prescribe for a patient, of course, we had the knowledge that first dose has to be taken at the facility, but some could say when one takes the dose with an empty stomach, they get weaker. We could give AL and advise patients to take at home. With the messages it was never the same, we emphasized that the patient takes it at the facility. I remember this other time; we got cups from… I think the ministry of health, cups [now missing] and a pail for water to ensure that all patients get their first dose right at the facility… AL does help, and we encourage people not to go before taking the first dose.* (Arm 1, Drug Dispenser)

Despite one of the SMS reminders stating that AL could be taken on an empty stomach, some HWs noted that they were hesitant to give AL to patients who appeared not to have eaten:

*We were giving the first dose at the facility but not all the times because this year there is famine and many patients were complaining that they did not eat. It was hard for us to give the first dose at the facility since the patients were looking weak. For those that were not weak, we were able to provide them with the first dose at the facility because we have cups at the pharmacy which people use to take drugs. Even if we notice that the child is very ill and ate something the evening before we could give them water right here to take the drugs and ask the participant to stay close to the pharmacies window to be observed. After observing that the child is fine we could ask the mother to go home, but some were given the first dose at the facility, and we noticed that they were becoming weaker. We could tell that it was a result of hunger.* (Arm 2, Drug Dispenser)

### Interactions between clinicians and drug dispensers

Some drug dispensers and clinicians reported incorrect dosing of AL that was based on the weight of the child. After seeing that the clinicians had given a wrong dosage to a child, a few drug dispensers discussed with their clinicians to change the dosage. Others were afraid that they would be labelled as ‘policing’ the in-charge, while other clinicians accepted the drug dispensers to act as check and balances on all IMCI drugs.

…*Yes. Some doctors understood, for example, doctors from under five were able to understand one day I returned the patients to the clinic because the information in the health passport book was missing. The child was coughing, and AL 6x4 was needed. I showed the SMS reminder to the doctor on duty, and they revised the prescription*. (Arm 1, Drug Dispenser)

*…text messages helped us a lot because sometimes most of the HWs’ (nurses) who come here are not experienced clinician but trainee doctors who usually come for practical so they don’t have more experience, for example sometimes they can over prescribe or under prescribe dose to a child without looking on weight and age of the child, but we were able to take part to communicate with them apart from being our seniors but reminding them the right dose.* (Arm 2, Clinician)

### Messages on severe malaria

All HWs mentioned the common symptoms of severe malaria which included fever, convulsions, vomiting everything, and anaemia. Most drug dispensers reported not having direct contact with patients suffering from severe malaria, but they knew case management guidelines. To manage severe malaria, most medical assistants reported that they prescribed a pre-referral drug, injectable artesunate, followed by referral to a district hospital. Other HWs mentioned using quinine as an alternative when artesunate was out of stock. However, there were some HWs who reported not adhering to severe malaria management guidelines due to drug stock-outs, caregiver’s request not to be referred, transport problems and living in a hard-to-reach area. (Quantitative data showed that 21/22 (95.5%) of the facilities that took part in qualitative interviews had either injectable artesunate or quinine available on the day of the survey (Steinhardt et al. pers. comm.). These HWs would treat cases of severe malaria with intravenous artesunate and AL at the health facility level and keep the children under-5 under observation until they were better.

*Malaria, mostly the management is like so… at first place, it depends on the medication we have as of now we have drug stock*-*outs, and we have transport problems, there is no ambulance, so most patients do not have money to go to the referral hospital. When patients stay close to the health facility, we advise them to come here and get their injectable drugs at intervals. When the patient is responding well, we do not admit. However, when they are not responding well to medication, I am forced to refer them to the district hospital for further management. There can always be some [alternative] medication which can be administered to them. However, for patients who stay far away, I still keep them under observation at the office until they are feeling better. Then they can continue with AL.* (Arm 2, Clinician)

### Messages on pneumonia and diarrhoeal diseases

For pneumonia-related ailments, both clinicians and drug dispensers were overall satisfied with the SMS messages they received. All HWs reported that SMS messages on pneumonia contained sufficient information to educate and remind them how to both diagnose and treat pneumonia, as most of them were not so familiar with the disease. However, despite the new knowledge reportedly gained through the SMS intervention, more HWs could recall malaria-only related messages than pneumonia or diarrhoea-related messages. HWs reported that this was due to fewer pneumonia and diarrhoea cases that presented during the intervention period.

Most HWs, especially drug dispensers, tensed up and seemed confused when responding to any interview questions relating to pneumonia. Likewise, some HWs said they failed to adhere to guidelines due to lack of practice as many children presented with a cough but only a few children with pneumonia reported to the facility. Others reported that they associated pneumonia symptoms with malaria, which caused the child to be misdiagnosed or left untreated. Many HWs complained that they faced challenges in observing the chest in-drawing, the breaths per minute using a timer and remembering the cut-off points according to children age groups, including providing the right prescriptions.

*On signs of pneumonia in children, there is nasal flaring, chest in*-*drawing and fast breathing; it is a main sign of pneumonia. We count the number of breaths per minute and refer to a chart; I do not remember the number of breaths, I did not memorize it* (IDI, Arm 2, Clinician).

HWs maintained that diarrhoeal diseases were the easiest to diagnose and treat in all study sites. Most of the HWs who received SMS reminders on diarrhoea seemed satisfied and were able to recall most of the specific messages. A few HWs complained that sometimes diarrhoea was over-treated with antibiotics due to stock-outs of zinc. A few (5/50, or 10%, claimed they were forced to use non-prescribed medicines to care for children with diarrhoea.

*However, the only challenge was the scarcity of drugs which hindered the following of the prescription from the texts. This was especially true in treating diarrhea. We have not had zinc for months, and so we use metronidazole and cotrimoxazole as antibiotics in place for zinc even though it is not recommended.* (Arm 2, Clinician)

### Sensitization, supervision and monitoring

Most HWs complained that they were not sensitized about the SMS intervention study. Others reported that they began to receive the SMS reminders without background information about the research study. On the other hand, most HWs claimed they received phone calls to confirm their participation in the study. There were mixed opinions among HWs on lack of supervision and monitoring exercises specific to the study to ensure support structures were available for study participants.

Some HWs criticized the lack of study-specific follow-up visits/supervisory visits including feedback sessions, as it made them feel as if “no one cares,” especially the implementing partners and the district health management team. The majority (45/50, or 90%) of HWs had expectations of attending either monthly or quarterly meetings as part of the intervention package, which would be linked to financial incentives. Both clinicians and drug dispensers were demotivated due to the lack of supervision and feedback sessions as narrated below.

*If there can be any changes maybe, we should be receiving phone calls. You should be coming after two or three months to supervise to find out whether the programme is working if the messages are helping and people are working accordingly.*

***Do you have anything to add towards this intervention?***

*Respondent: The receivers of the messages should have time to meet each other or have workshops.*

***Probe: What other additions do you have?***

*Respondent: We were told that we would be receiving messages for free, just to be encouraged. You have inspired us, we are working accordingly, unlike some pharmacies who only give injections and people get satisfied. You should be calling us and seeing if we are doing well, examining us on this.* (Arm 1, Drug Dispenser)

*Incentives can come later after supervision. We need supervision to know that we are performing well. We need to be encouraged. Incentives* *come secondly, but the most critical thing is supervision.* (Arm 2, Medical Assistant)

On an individual level, a few clinicians discussed their performance including monitoring malaria drug use with the help of drug dispensers at the facility level. Some clinicians and medical assistants became change agents in their health facilities, as they were able to present or share SMS reminders during morning briefs. Others claimed the SMS intervention study helped to change both themselves and newly recruited team members in their facility. Others believed the SMS intervention study was part of ‘virtual monitoring’ by the National Malaria Control Programme. HWs were encouraged to adhere to guidelines to please the ministry. HWs reported that behaviour change was readily noticed when they tried to use the SMS reminders to improve diagnostics and adhere to treatment guidelines.

*As I have already said that after receiving the message, I could go and share with all the clinical officers, medical assistants and nurses, I could read the message to them during handovers in the morning (morning briefs) and tell them what could be done and what not to do. They were responding very well; things were changing since I am the in*-*charge for the OPD. I had so many challenges with the interns from the school regarding the prescription. After sharing with them the SMS reminders, there was much improvement.* (Arm 2, Clinician)

### Alternative messaging

Most HWs did not propose alternatives to the SMS intervention study. They were in favour of maintaining the SMS reminders because phones are personal and very convenient reference materials. A few HWs suggested that implementers consider adding SMS reminders on other common illnesses to provide a more comprehensive package, e.g., expanding malaria, diarrhoea, pneumonia to asthma and skin problems. Other HWs talked of having pamphlets or leaflets that could be referenced easily on their desk. A few HWs asked for short handbooks that could be easily accessed by other team members and are easier to share with colleagues than continuously giving out a personal phone. Posters were also suggested as they could be easily accessed by caregivers, hence providing more space for reading them on the walls at the clinic. A few people also suggested the internet and radio reach out to the masses and involve the community.

## Discussion

This study examined the acceptability and feasibility of using SMS reminders to improve HW adherence to malaria, pneumonia and diarrhoea case management guidelines. The HWs generally viewed the SMS intervention favourably and accepted the SMS as job aids and practical reference material for their day-to-day work. A previous study that sent malaria SMS case management reminders to HWs in Kenya also found high acceptability among HWs receiving messages [22], echoing other studies using SMS reminders either to improve HW adherence to guidelines [14, 17, 23, 24] or patient adherence to medications [25].

Concerning the modalities of SMS intervention, HWs were supportive of the timing of messages received, their frequency, length, and language used. The repetition pattern was, however, less appreciated among HWs receiving only malaria messages compared to those HWs who were exposed to the greater variety of messages, which included management of pneumonia and diarrhoea in addition to malaria. Notably, despite some concerns about the repetitive patterns, nearly all HWs expressed a wish for a longer duration of the SMS intervention including other diseases.

The trial in Malawi revealed, however, limited improvements in case management practices for the targeted diseases (Steinhardt et al., pers. comm.), in contrast to what has been observed in another study focusing on malaria in Kenya [14]. The qualitative investigations reported in this paper elucidated several, mainly health systems barriers, as to why the SMS might not have achieved their intended impact. First, despite understanding and appreciating the importance of the messages, HWs promptly referred to commodity stock-outs as one of the major barriers influencing adherence to guidelines. For instance, lack of cups and clean water obviously preclude prompt treatment at facilities. Similarly, there is widespread evidence in Kenya, Tanzania and Malawi on the role of health systems barriers in curtailing advances in technology for health or treatment services [11, 23, 25, 26].

While commodity stock-outs only partly explain the problem of non-adherence, as drug stocks were generally good at these facilities, outpatient workload were mentioned throughout the interviews as a significant barrier, especially for the performance of more time-consuming tasks such as counting of respiratory rates, testing of all malaria-suspected patients, or administration of the first dose at the health facility. Lower outpatient caseloads have been associated with better HW performance in other settings [27–29]. The fivefold lower caseload observed in the Kenyan SMS study setting, the focus of the intervention on rural dispensaries staffed by nurses, and continuous availability of commodities at the study facilities in Kenya were likely factors contributing to the case-management improvements observed in the Kenyan but not in the Malawian trial.

HWs showed greater awareness about malaria than pneumonia recommendations, and could not often recall the pneumonia messages during the interviews despite repeatedly sent messages. The study context in which the SMS reminders followed an in-service malaria training with high coverage, but a lack of IMCI training and supportive supervision focused on IMCI in the last several years may have contributed to the observed pattern [30, 31].

Lack of follow-up visits, supervision and feedback either through mobile technology or through in-person meetings, which is often associated with financial incentives in Malawi, was another barrier dominating HWs’ responses when explaining non-adherent practices. Ongoing supportive interventions have been shown to be useful in reinforcing HW practices [11, 26]. Despite these SMS reminders being designed as malaria post-training support, lack of integration of SMS case-management standards into routine supervision is a missed opportunity to strengthen not only SMS intervention but also supervisory visits. Whether more interactive, but more complex-to-implement, two-way technology communication systems, including supervision, may be more effective in resolving some of the health systems barriers and improve adherence to guidelines, as suggested in other settings [11, 16, 23] remains to be shown in Malawi. Results from this study show that SMS reminders, without additional human or financial resources to support their implementation, for example, through enhanced supervision on the SMS, might not be effective in improving case management in routine clinical settings with high patient volume settings such as Malawi.

Finally, despite the simplicity of distributing electronic bulk messages to a large number of recipients, this study found that many of the HWs targeted for interviews had moved or were no longer in their position, resulting in low coverage of HWs: approximately 46% receiving message (Steinhardt et al, pers. comm.). This was substantially lower compared to over 90% of exposed HWs after a year of the trial in Kenya [14]. While no evidence of better performance was observed among exposed HWs (Steinhardt et al, pers. comm.), overall low exposure saturation may have been another factor contributing to the lack of effect observed in Malawi but not in Kenya.

## Limitations

This study had several limitations. Data collected relied on individual reports, which are subject to recall bias and social desirability on issues challenging HW professional autonomy. Also, a sizeable proportion of the HWs targeted for the interview were transferred to another facility or returned to school, and their perceptions were not able to be captured.

## Conclusions

mHealth has the potential to support HWs in case management practices, but health systems barriers related to commodity stock-outs, high workload, and lack of ongoing support interventions need to be addressed prior to or alongside implementation of reminder interventions to improve quality of care. Although HWs might possess knowledge, they might not always put it into practice, a finding in line with that of a systematic review that found no association between knowledge and clinical practice [32]. For the success of SMS interventions among HWs, it is recommended that programmes develop more interactive delivery methods, such as facilitating teamwork among health facility staff, and consider structural barriers that might prevent full implementation of SMS reminders, along with supervisory support to improve case management for patients in low-resource settings [12, 14].
